# More Than a Moggy; A Population Genetics Analysis of the United Kingdom’s Non-Pedigree Cats

**DOI:** 10.3390/genes12101619

**Published:** 2021-10-14

**Authors:** Jennifer Irving McGrath, Wengang Zhang, Regina Hollar, Alison Collings, Roger Powell, Rob D. Foale, Nicola Thurley, Jeffrey A. Brockman, Richard J. Mellanby, Danièlle A. Gunn-Moore, Jeffrey J. Schoenebeck

**Affiliations:** 1 Royal (Dick) School of Veterinary Studies & Roslin Institute, University of Edinburgh, Easter Bush Veterinary Campus, Midlothian, Edinburgh EH25 9RG, UK; s0782801@ed.ac.uk (J.I.M.); wengang.zhang@ed.ac.uk (W.Z.); Richard.Mellanby@ed.ac.uk (R.J.M.); danielle.gunn-moore@ed.ac.uk (D.A.G.-M.); 2 Hill’s Pet Nutrition Centre, Topeka, KS 66603, USA; regina_hollar@hillspet.com (R.H.); Jeff_brockman@hillspet.com (J.A.B.); 3 Idexx Laboratories, Wetherby LS22 7DN, UK; Alison-Hall@idexx.com; 4 DragonVet Consulting Ltd., Hertfordshire G5 1EH, UK; vetpath@dragonvetconsulting.co.uk; 5 Dick White Referrals, Station Farm, London Road, Six Mile Bottom, Cambridgeshire, Cambridge CB8 0UH, UK; rf@dwr.co.uk (R.D.F.); nikki.thurley@dwr.co.uk (N.T.)

**Keywords:** cat, population genetics, structure, SNV, genotype, random-bred, inbreeding, autozygosity

## Abstract

The domestic cat is one of the most popular pets in the world. It is estimated that 89–92% of domestic cats in the UK are non-pedigree Domestic shorthair (DSH), Domestic longhair (DLH), or Domestic semi-longhair cats (DSLH). Despite their popularity, little is known of the UK non-pedigree cats’ population structure and breeding dynamics. Using a custom designed single nucleotide variant (SNV) array, this study investigated the population genetics of 1344 UK cats. Principal components analysis (PCA) and fastSTRUCTURE analysis verified that the UK’s DSH, DLH, and DSLH cats are random-bred, rather than admixed, mix breed, or crossbred. In contrast to pedigree cats, the linkage disequilibrium of these random-bred cats was least extensive and decayed rapidly. Homozygosity by descent (HBD) analysis showed the majority of non-pedigree cats had proportionally less of their genome in HBD segments compared to pedigree cats, and that these segments were older. Together, these findings suggest that the DSH, DLH, and DSLH cats should be considered as a population of random-bred cats rather than a crossbred or pedigree-admixed cat. Unexpectedly, 19% of random-bred cat genomes displayed a higher proportion of HBD segments associated with more recent inbreeding events. Therefore, while non-pedigree cats as a whole are genetically diverse, they are not impervious to inbreeding and its health risks.

## 1. Introduction

The domestic cat is the one of the most popular pets in the world. There are an estimated 106.4 million pet cats in Europe, 95.6 million pet cats in the United States of America (USA) [1], and the United Kingdom (UK) alone has 10.9 million pet cats [2]. It is estimated that non-pedigree cats make up the vast majority of this population, with only 8–11% of owned cats in the UK being pedigree cats [3,4].

Evidence presented by both Lipinski et al. [5] and Kurushima et al. [6] proposed that pedigree cats continue to share genetic ancestry with the cats local to these breeds’ geographic origins. Thus Siamese, Birman, and Burmese cats share genetic ancestry with random-bred cats found in Asia, while the Persian, British shorthair, and Maine coon cats align with random-bred cats living in Western Europe and the Americas [5,6]. Random-bred cats are the populations from which many early breeds were developed and “Modern” cat breeds emerged from early breeds, typically as coat colour and hair variants of the latter [7], for example, Exotic cats are the short-haired variant of the Persian breed [8]. Today, there are many cat registries that represent domestic and international interests. The oldest is the Governing Council of Cat Fancy (GCCF, UK) which currently recognises 41 breeds. The Cat Fanciers’ Association (CFA, USA) recognises 45 breeds and the International Cat Association (TICA) recognises 71 breeds.

Cats that are not pedigree are known by various monikers including “moggy”, “outbred” and “crossbreed”, to name a few. Non-pedigree cats represent the vast majority of pet cats; in the UK, they are estimated to represent ~90% of its owned feline population [3,4] and almost all of the feral cats. Previous feline population genetics studies [5,6,9] have called non-pedigree cats ‘random-bred cats’, in reference to the fact there are no specific breeding policies or registries in place for these cats. Random-bred cats are distinct from mixed or cross breeds, as the latter names suggest derivation from pedigree animals. The majority of random-bred cats in the UK are classified as either a Domestic shorthair (DSH), Domestic longhair (DLH), or Domestic semi-longhair (DSLH) cats depending on their coat length. These cats are a medium sized cat with a proportional body type and no extreme skeletal or facial features. With the exception of soft curly fur (e.g., “rex”) or almost no fur (e.g., “Sphynx”), all coat types and patterns are recognised. Coat length in domestic cats is determined by autosomal recessive mutations in the *FGF5* gene [10,11]. Few DSH, DLH, and DSLH cats are selectively bred; instead, they pick their own mates from other local DSH, DLH, and DSLH cats.

Despite the majority of pet cats in the UK being non-pedigree cats, most genetic mapping studies of heritable conditions investigated pedigree cats. The reasons for this are obvious: pedigree animals tend to be less genetically diverse due to line breeding, use of popular sires and historic bottlenecks; presumably their inbreeding exposes simple Mendelian disorders. Moreover, the availability of pedigree information, which is usually absent in non-pedigree cats, facilitates study design by revealing inheritance (i.e., recessivity, penetrance, disease risk).

The current commercially available Illumina Infinium iSelect 63 K feline single nucleotide variant (SNV) array has successfully mapped numerous feline traits and Mendelian diseases [9,12,13,14,15,16,17]. The markers used on this array were discovered through resequencing an assortment of pedigree cats and wild felids to supplement the markers discovered from the production of the Abyssinian cat genome assembly. Whilst the 63 K array has proven its utility for population studies of pedigree cats, the density of markers was predicted to fall short of that needed to study random-bred cats. The increased genetic diversity and, by extension, shorter haploblocks of these populous cats, necessitate higher marker density to achieve genome wide coverage.

Due to the immense popularity of the UK’s non-pedigree cats, the primary aim of this study is to understand their genetic structure and contrast them against local pedigree animals. Assessments utilised custom arrays designed by Hill’s Pet Nutrition to interrogate 340 K single nucleotide variants (SNVs), more than 5 times the density of the current 63 K SNV array. As the array was based on marker discovery from 6 DSH cats, a second aim of this study was to assess the Hill’s array in its ability to discern differences between random-bred cats and pedigree cats. This study serves as a foundation to future population studies, for example, genome-wide association of diseases observed among the U.K’s random-bred cats.

## 2. Materials and Methods

### 2.1. Study Participants

In total, 1344 domestic cats were recruited from the Royal (Dick) School of Veterinary Studies (R(D)SVS) Feline Biobank. This biobank is populated with blood, saliva, or genomic DNA samples submitted by the R(D)SVS Hospital for Small Animals, Dick White Referrals, Davies Veterinary Specialists, IDEXX laboratories (Wetherby, UK), other UK referral hospitals, and various general practices throughout the UK. Inclusion criteria included the cat’s breed (if applicable) and gender as reported by either the owner or veterinary professional. Ethical approval was obtained from the R(D)SVS Veterinary Ethical Review Committee (approval numbers 127.16 and 47.17) and owner consent was obtained for use of their cat’s data in research.

### 2.2. Samples, DNA Extraction and Genotyping

Biological samples used for genomic DNA (gDNA) extraction included the residual volumes of whole blood in EDTA following completion of diagnostic tests, saliva swabs, or *post-mortem* muscle biopsies. No samples were taken specifically for this study. gDNA was extracted from residual whole blood in EDTA samples (stored at 4 °C, −20 °C or −80 °C) [18]. Saliva samples were taken using DNA Genotek PERFORMAgene kits and stored at room temperature until extraction. gDNA was extracted from kits using the manufacturer’s protocol [19]. *Post mortem* muscle biopsies, stored at either −20 °C or −80 °C, were extracted as described by Marchant et al. [18]. All gDNA samples were gently resuspended in TE buffer and stored at 4 °C. Following resuspension, gDNA was first quantified using Nanodrop [20]. Samples selected for genotyping were then assayed using either Qubit [21] or Picogreen assay [22].

Samples with at least 600 ng of DNA were selected for genotyping by Edinburgh Genomics, using custom Illumina Infinium iSelect Beadchip arrays (90 K and 250 K) designed by Hill’s Pet Nutrition. The arrays contain 340,000 attempted beadtypes for genotyping single nucleotide polymorphisms selected across the entire cat genome using the feline genome assembly *Felis Catus* 6.2. SNVs for the array were selected from whole genome sequencing of 6 genetically diverse female domestic shorthair cats. The cats were sequenced on a HiSeq2500 (Illumina, San Diego, CA, USA) to generate 100 bp paired-end reads. Following GATK best practices pipeline [23], reads were mapped to the feline reference genome using BWA mem [24]. Duplicate reads were tagged by PICARD [25] mark duplicates and indels were realigned and quality scores recalibrated using GATK. Variants were called and filtered using GATK HaplotypeCaller and VCFtools [26]. The full list of variants was thinned randomly using PLINK [27]. Protein coding SNVs predicted to have a moderate or high impact by SNPEff [21] were added to the beadpool.

### 2.3. Genotype Analysis

Genotypes were remapped to *Felis Catus* 9.0 as the SNV arrays were designed on a previous cat genome version (*Felis Catus 6.2*). A three-step pipeline was developed to remap the 340,000 SNVs. Firstly, the probe sequences were aligned against *Felis Catus 9.0*. The command: *blastn -db Felis_catus_9.0 -query cat_90K_100bp.fsa -max_hsps 3 -evalue 1e-30* was used to map 101 bp flanking sequence of each probe from *Felis Catus* 6.2 to *Felis Catus* 9.0. Secondly, multiple mapped probes and questioned probes were filtered out, retaining only probes that had one mapped position or that matched one subject perfectly. Finally, golden probe positions were fixed in the *Felis Catus* 9.0 genome with the following strategy: (1) if the alignment had no gap, we inferred the designed SNVs’ positions by coordinates, (2) if the gap was >0, we collected 50 bp of proximal and distal flanking sequences surrounding each SNV and re-aligned it to *Felis Catus* 9.0. (3) For each SNV probe with a gap, if the inferred SNV position based on the alignment of the proximal 50 bp sequence was the same as the inferred SNV position based on the alignment of the distal 50 bp sequence, the inferred position was considered reliably remapped.

After remapping, 39,757 variants were filtered out due to either multiple mapped positions or due to unstable positioning as a result of SNV proximity to indel(s) or simple repeat elements, leaving 263,482 variants in our raw dataset.

Genotype quality control analysis was performed using PLINK v1.90b [28,29]. Duplicate samples were flagged and the sample with the least missing data was retained. SNVs with a genotyping rate of 80% or less were removed using the plink command “*--geno*”. Samples with more than 50% missing genotype data were removed using the plink command “*--mind*”. The remaining SNVs were then filtered again to remove SNVs with a genotyping rate of less than 98%. Of the remaining samples those with a genotyping rate of less than 95% were removed. Breed specific Minor Allele Frequencies (MAF) were then calculated using the commands “*--freq --family*”. Observed Heterozygosity (H_o_) was calculated using “*--hardy*”. The Inbreeding coefficient (F_is_) and observed homozygosity was calculated using *“--het*”. Finally, only SNVs with a MAF ≥ 0.05 were retained using the plink command “*--maf*”.

### 2.4. Population Ancestry and Structure Analysis

**Figure A1:**
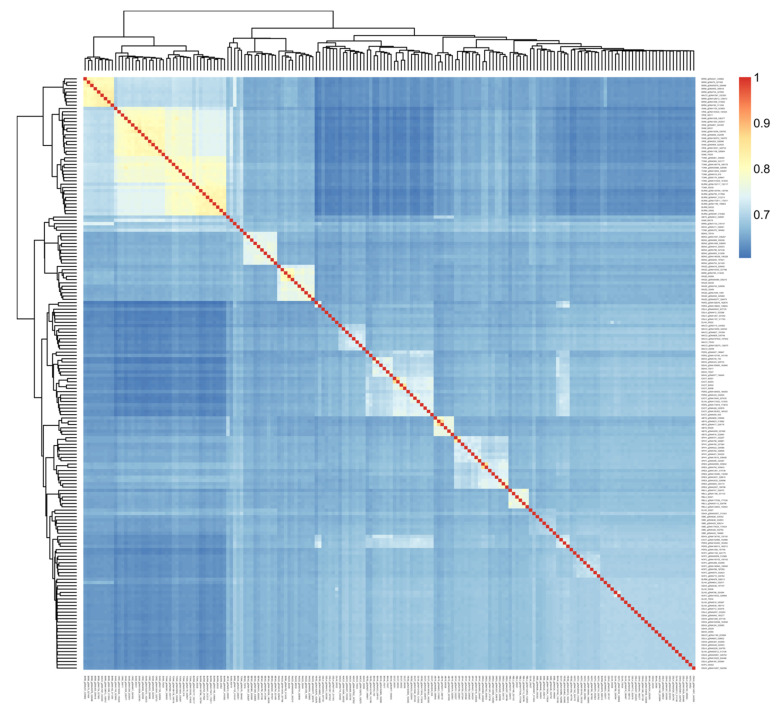
IBS matrix of the balanced data ordered by hierarchical clustering.

**Figure A2:**
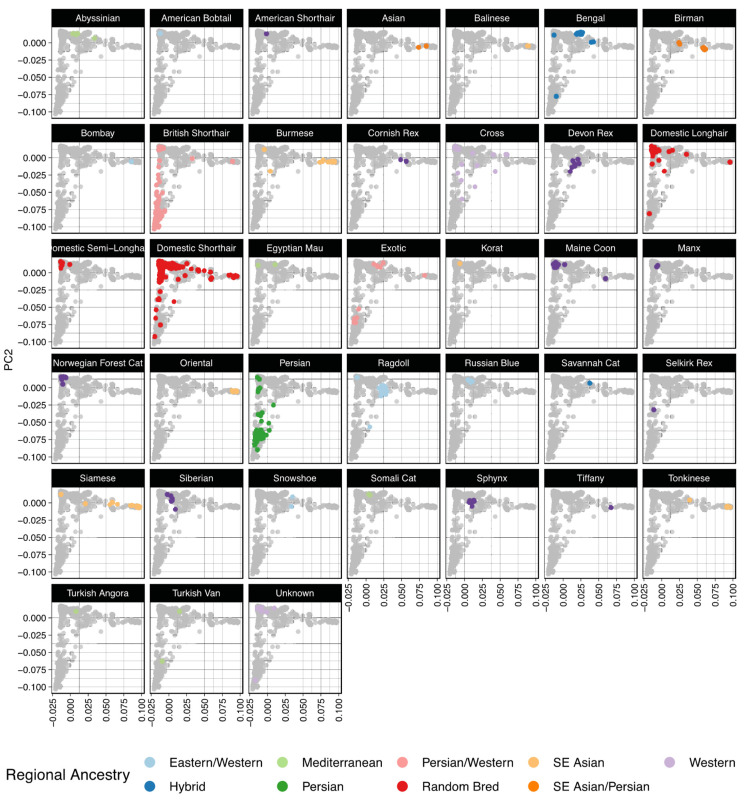
Principal components of the full dataset coloured by breed/group.

Population structure analysis was conducted on an autosomal dataset that had been pruned for variant pairs in Linkage Disequilibrium (LD) using the command *“--indep-pairwise 50 5 0.5*” in PLINK v1.90b [28,29]. Principal Component Analysis (PCA) was also conducted using PLINK v1.90b. Using the option “*--pca*”, the top 20 principal components of the variance-standardized relationship matrix were extracted. The components were plotted using the package “ggplot2” [30] in R, version 3.5.1 [31] in R Studio, version 1.1.456 [32]. Structure and admixture were assessed using fastSTRUCTURE; the “--chooseK” tool was used to find the appropriate K level. [33]. Because the large disparity in breed sampling numbers confounded interpretation, breeds with fewer than 5 samples were not considered further. A “balanced dataset” was produced containing breeds with more than 5 samples and for those breeds with more than 10 samples, the R function “sample_n” was used from the dplyr package [34] to select 10 cats at random from each breed. Similarly, 10 DSH, 10 DSLH, and 10 DLH were selected at random for downstream comparisons using the balanced dataset. Cryptic relatedness was evaluated by generating an IBS matrix using the flags “*--distance square ibs*”. A heatmap (Figure A1) was produced from the matrix using the R “pheatmap” package (v1.0.12). PCA was conducted on the balanced dataset and also on three additional balanced datasets (Figure A2) to ensure general PCA trends were consistent with our random sampling. LD pruning was conducted on the primary balanced dataset using the previously mentioned command, fastSTRUCTURE results were plotted using Clumpak [35] for the balanced dataset and the R package “Pophelper” [36] in R Studio, version 1.1.456 for the complete dataset.

### 2.5. Linkage Disequilibrium Analysis

Genome-wide LD was estimated for the balanced dataset using the squared correlation coefficient (R^2^) between pairs of SNVs as implemented in PLINK v1.90b. Analysis on variants up to 4000 kb apart or with up to 99,999 SNVs between them was reported for each breed individually. Results were then plotted in relation to other breeds using R Studio version 1.1.456.

### 2.6. Homozygosity by Descent

Homozygosity by descent (HBD) was assessed using the R package “RZooRoh” [37]. This software identifies HBD segments associated with regions of homozygosity (ROH) and is based on a hidden Markov model framework. Genome-wide individual autozygosity is partitioned into different age-related HBD classes under the assumption that each class has its own length and frequency. The different HBD classes are defined by their specific rates R_k_, where k is the class number. Classes with lower rates are associated with longer HBD segments from more recent common ancestors. Therefore, different HBD classes can be interpreted as HBD segments from successively older generations [37]. The zoomodel function was used to define a model, with 10 classes (9 HBD and 1 non-HBD class) with a ratio between successive rates of two (i.e., *R_k_* equal to 2, 4, 6, 8, 16, 32, 64, 128, 258, and 512). The zoorun function estimates the parameters of the model, the global and locus specific realized autozygosity, to partition it in the different HBD classes and to identify the HBD segments. A maximum of 100 iterations of the EM-algorithm were selected for this function and convergence criteria for the EM algorithm was set at 1 × 10^−12^.

## 3. Results

Post genotyping, the quality control process resulted in an analysis-ready genotypes consisting of calls from 1290 cats and 178,506 SNVs (Table 1).

These genotypes were from 33 breeds; DSHs, DLHs, and DSLHs; a group of cross-bred cats (e.g., registered as Persian cross or Siamese cross) and the unknown group (genotyped for a separate study) as presented in Table 2. The average MAF across all breeds prior to MAF filtering was 0.25 (range 0.24–0.27). Breeds considered Western in origin such as the Persian had slightly lower average MAF than Eastern origin breeds such as the Siamese (average MAF 0.24 and 0.27, respectively). Average observed heterozygosity (H_o_) prior to MAF filtering across all breeds was 0.25 (range 0.18–0.32). Eastern breeds including the Korat and the Burmese showed the least heterozygosity (H_o_ = 0.18 and 0.19, respectively), with the American Bobtail and the American shorthair having the highest (average H_o_ = 0.32); however, both breeds had only one representative in the dataset. The DSH and the Norwegian Forest cat are also worth noting as they had a higher H_o_ at 0.3. The inbreeding coefficient (F_is_) ranged from −0.017 in the Abyssinian and Exotic shorthair to 0.113 in the Persian. The DSH, DLH and the British shorthair had the least monomorphic SNVs at 8%, 10%, and 11% respectively, whilst the Korat, Bombay and Tiffany had the highest at 82%, 80%, and 76%, respectively.

### 3.1. Population Ancestry and Structure

**Figure 1:**
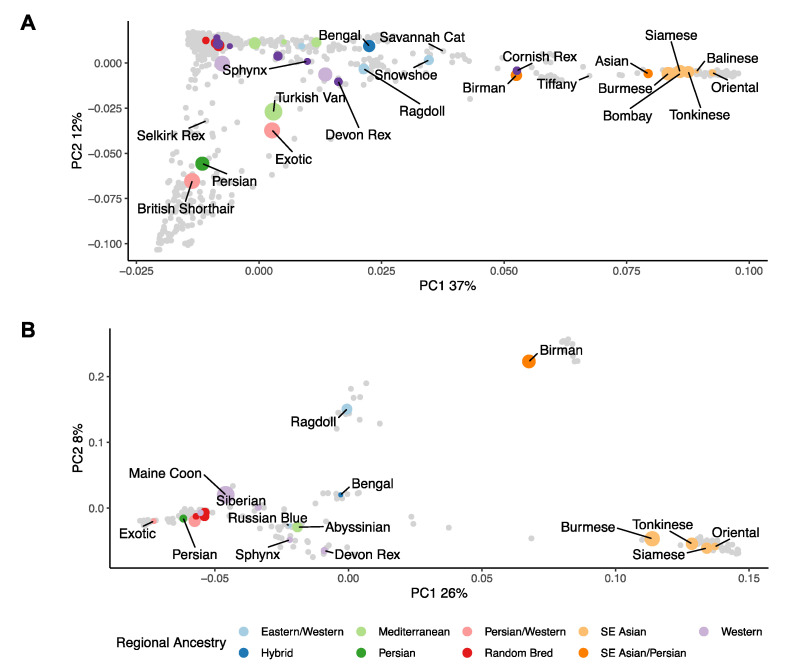
Principal component analysis (PCA). (**A**) PCA analysis for all 1290 samples. (**B**) PCA analysis for all samples in the balanced dataset. A subset of breeds is labelled to avoid overplotting.

**Figure A3:**
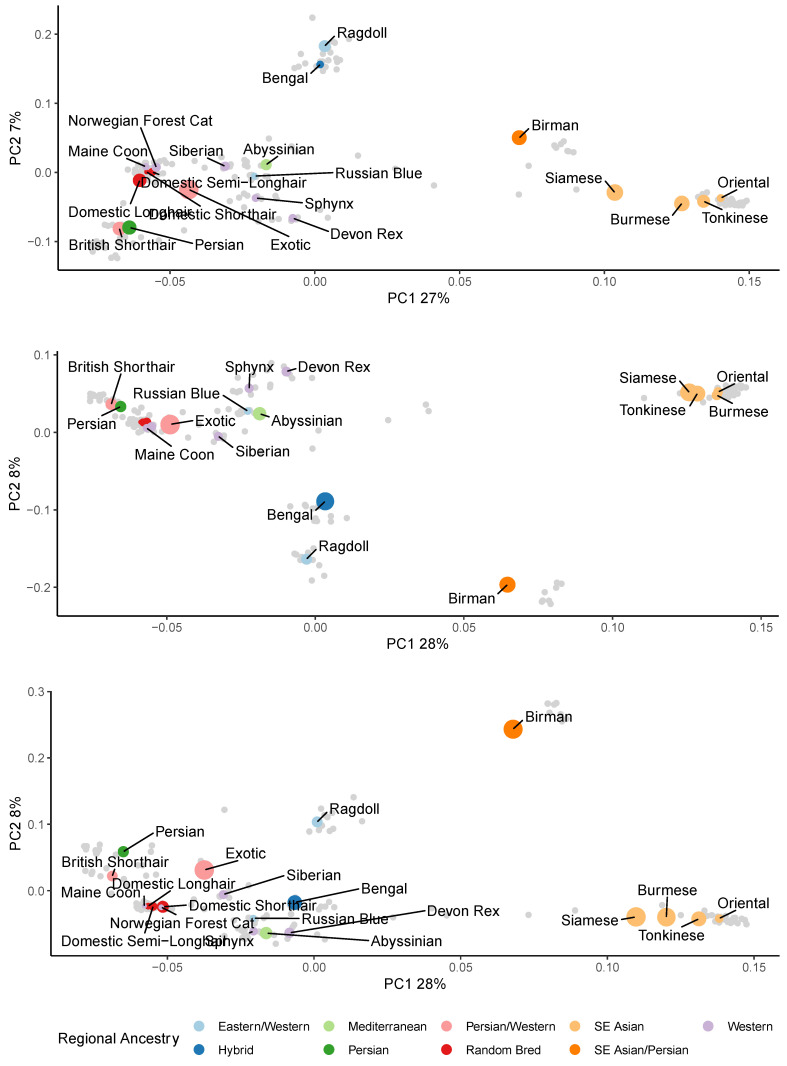
PCA for three additional balanced datasets (randomly chosen). Comparison of panel A to other panels’ PC2 axes reveals instability in the ancestry described by this PC.

To infer ancestry between all 1290 cats, principal component analysis (PCA) was used to decompose the genetic matrix. LD pruning resulted in 132,240 remaining SNVs for population structure analysis. Forty-nine percent of genetic variance is explained by the first two components; PC1 explains 37% of the variance whilst PC2 explains 12% (Figure 1A). PC1 divided Western derived and Southeast Asian breeds. Owing to ascertainment bias in array design, most DSH, DLH, and DSLH cats clustered together and were polarised with respect to Southeast Asian and Persian breeds (Figure A3). The majority of DSH, DLH, and DSLH cats aligned with Western breeds such as the British shorthair and Persians on PC1. The British shorthair, Persian and Exotic breeds were separated from the majority of other cats by PC2.

With the potential for the uneven sampling to distort the analysis, we also looked at the ancestry of subpopulations represented by 5–10 cats each (hereafter referred as the “balanced dataset”, see Materials and Methods). The balanced dataset had a population size of *n* = 180 with 17 pure bred populations; the 3 random-bred populations and those breeds which had at least 5 cats sampled. Table 3 details those cats in the balanced dataset. After LD pruning 127,477 SNVs remained in the balanced dataset for population structure analysis.

**Figure A4:**
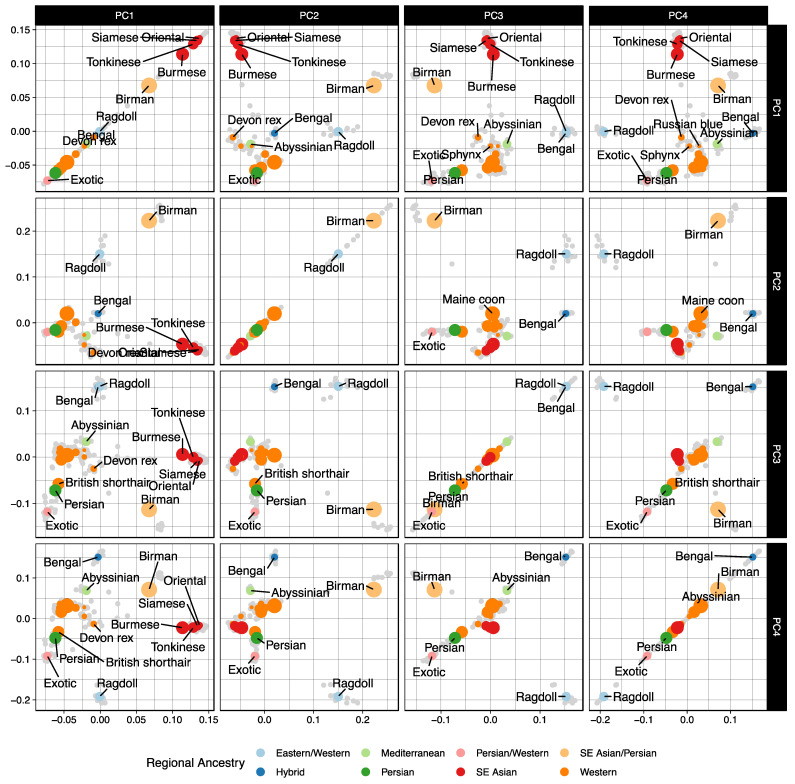
Combinations of PC1-PC4 for the balanced dataset. Coloured points reflect regional ancestry while the circle size reflects the maximal standard deviation across the depicted PCs.

Viewing the principal components of the balanced dataset, the ancestry between cat subpopulations is more apparent (Figure 1B). As before, PC1 still separates Southeast Asian and Western derived cats. However, PC1 describes less variance than it did in the full dataset (26% vs. 37%, respectively). PC2 (8%) polarised the Birman, and to a lesser extent, Ragdolls, with respect to other cat varieties. This observation is consistent with Gandolfini et al. [9], however its interpretation is unclear. Furthermore, when additional randomly sampled, balanced datasets were analysed by PCA, the position of varieties appeared relatively stable (Figure A3). With down sampling, the study observed that in the Birman cluster there is a cat whose records reported it as a Maine coon. Similarly, in the Ragdoll cluster there are two cats recorded as Birmans (data not shown). This may be due to reasons such as incorrect breed assignment or inaccurate clustering. Apart from two DLH, the DSH, DLH, and DSLH cluster tightly together with Western breeds such as the Maine coon and Norwegian forest cat, plus the British shorthair and Persians. Other combinations of PC1-PC4 can be found in the Appendix A (Figure A4).

**Figure 2:**
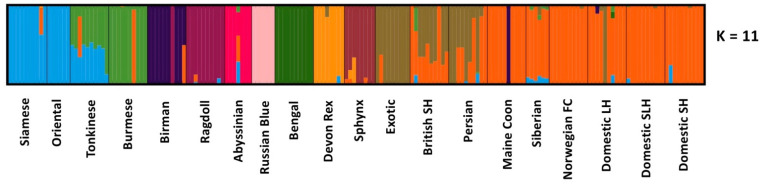
fastSTRUCTURE analysis on the balanced dataset. Each column represents an individual cat. Each cluster (K) is represented by a unique colour. The model component used to explain structure in the dataset (*K*∅*c*) was K = 11.

**Figure A5:**
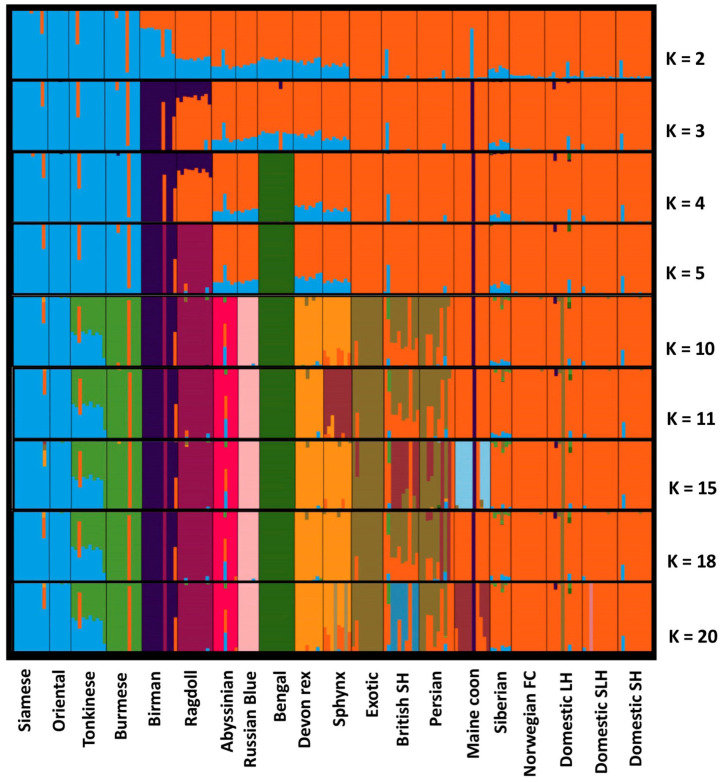
fastSTRUCTURE analysis on the balanced dataset. Each column represents an individual cat. Each cluster (K) is represented by a unique colour. The model complexity maximizing marginal likelihood was K= 10. Model components used to explain structure in the dataset (*K*∅*c*) was K = 11. This suggests that 11 is the minimum number of populations in the balanced dataset that have a cumulative ancestry contribution of at least 99.99%.

Population structure was also investigated using the variational Bayesian framework implemented by fastSTRUCTURE [33]. Analysis from K = 2 to K = 30 was conducted on both the balanced dataset and the full dataset. Analysis by fastSTRUCTURE using the balanced dataset produced a more defined output with a model complexity maximizing marginal likelihood of K = 10 and K = 11 for model components used to explain structure in the data (*K*∅*c* statistic in fastSTRUCTURE). This suggests that K = 11 is the minimum number of populations that have a cumulative ancestry contribution of at least 99.99% (Figure 2 and Figure A5). At K = 2, Southeast Asian breeds like the Oriental and Siamese separated, with other breeds following suit with increased K (Figure A5). At K = 11, Siamese and Oriental cats were not resolved; this is not unexpected as the Oriental cat is a Siamese in all but colour. It was developed in the 1950s, when Siamese were crossed with different cats including Russian blues, British shorthairs, DSH, and Abyssinians, then crossed back to Siamese cats [39]. The Ragdoll also has its own cluster and there appears to be a cat from the Ragdoll cluster in the Birman group (the same cat also evidenced in the PCA data). The Tonkinese are very clearly admixed with Siamese and Burmese, as expected given their derivation from these breeds [40]. DSH, DLH, and DSLH could not be resolved from the Maine coon, Siberian, and Norwegian forest cats. Admixture with these cats appears elsewhere, notably among Persian and British shorthair (who by PCA cluster closely with the DSH, DLH, and DSLH in our balanced dataset PCA). Regardless of K tested for the balanced dataset, DSH, DLH, and DSLH cats remained homogeneous (Figure A5).

**Figure 3:**
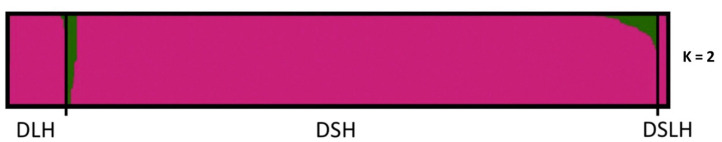
fastSTRUCTURE analysis of 840 DSH, DLH, and DSLH cats. At K = 2, 91% (*n* = 772) random-bred cats had little evidence of admixture. Model complexity that maximized marginal likelihood was K = 2, and the model components used to explain structure in the data were K = 2.

**Figure A6:**
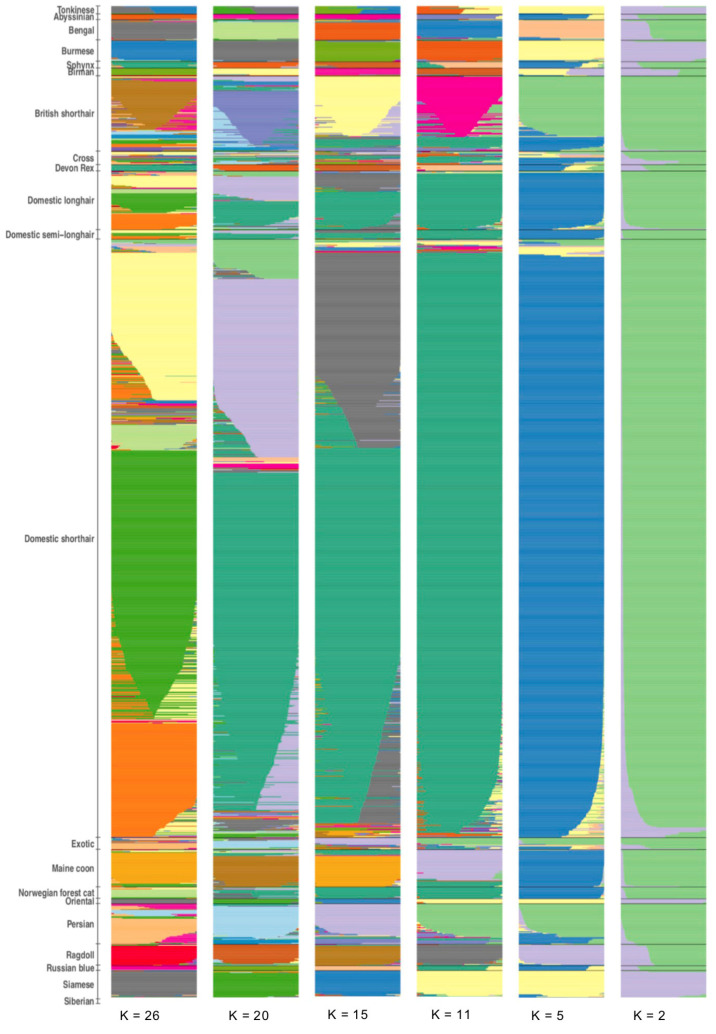
FastSTRUCTURE analysis of the full dataset (showing only breeds with 10 or more samples for display purposes). The model complexity that maximised marginal likelihood was K = 26, whilst model components used to explain structure in the data were K = 11.

Exploring further, fastSTRUCTURE was conducted on all 840 non-pedigree cats by themselves. Model complexity that maximized marginal likelihood and data structure were set at K = 2 (Figure 3). The overwhelming majority (91% of 840 DSH, DLH, and DSLH cats) have a proportion of ≥0.9 for one of the two clusters. fastSTRUCTURE was then run on the full dataset. This gave a broadly defined structure range with the model complexity that maximised marginal likelihood at K = 11 whilst the model component used to explain structure in data was K = 26 (Figure A6). This variation between the two parameters for choosing model complexity may be due to the large number of DSH samples dominating the dataset. However, at K = 26 in the full dataset, the DSH, DLH, and DSLH were resolved from the Maine Coons, the Norwegian forest cats, and the Siberians. Notably, 3 subdivisions within the DSH, DLH, and DSLH populations emerged at K = 20. As all 3 clusters contained samples originating from both England and Scotland, the partitioning of random-bred cats breeds is not explained by geography.

Together, PCA and fastSTRUCTURE point to a general uniformity for the UK’s DSH, DLH, and DSLH cats that is uncharacteristic of a crossbred or recently admixed population. Rather, the data indicate that the UK’s non-pedigree are mostly random-bred.

### 3.2. Linkage Disequilibrium

**Figure 4:**
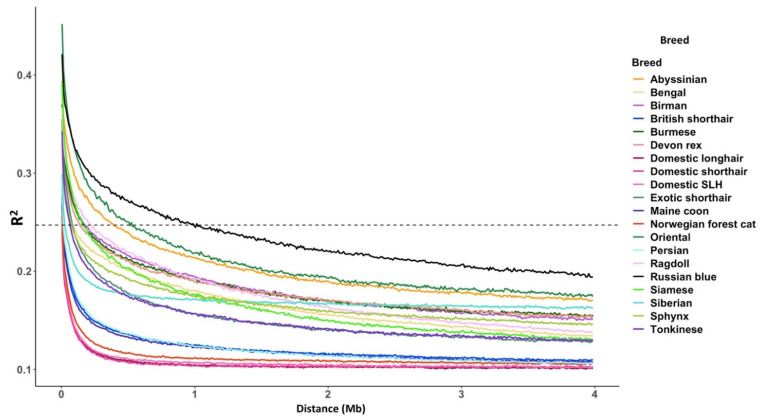
The genome wide rate of linkage disequilibrium decay. The dashed line marks R^2^ = 0.247 the maximum R squared in the random-bred population.

Decay of LD is typically rapid among outbred populations. To assess this, the study used the balanced dataset (Figure 4). Overall, the random-bred cats, DSH, DLH, and DSLH had the most rapid rates of LD decay, followed closely by the Norwegian forest cat. In contrast, the Oriental, Russian blue, and Abyssinian had the slowest rates of LD decay. Following the Norwegian forest cat’s rapid LD decay were the British shorthair, Persian, and Maine coon cats. The Siberian differed from other breeds in that LD initially decayed rapidly to below R_2_ = 0.17 within 1 Mb, but then remained relatively stable thereafter. This observation is perhaps reflective of a breed in the making: in its native Russia, Siberian cats are likened to an old regional variety of random-bred cats. Elsewhere, this cat is rare, and its selective breeding is a recent phenomenon; the first Siberian cats officially reached the UK in 2002; since then the number of UK breeders has increased rapidly [41].

### 3.3. Homozygosity by Descent

**Figure 5:**
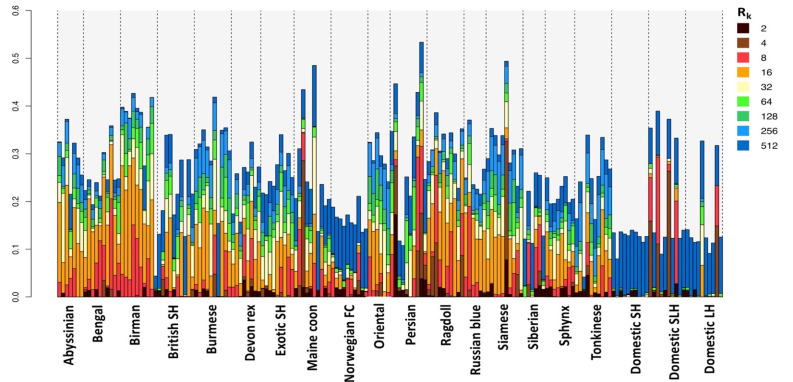
Genomic content of homozygosity by descent (HBD) segments. HBD segments were classified by segments’ ages with respect to inferred generation time. The colour legend indicates the HBD class rates, defined by R_k_. The rate of a class is equal to twice the number of generations to the common ancestors associated with that class [37].

**Figure 6:**
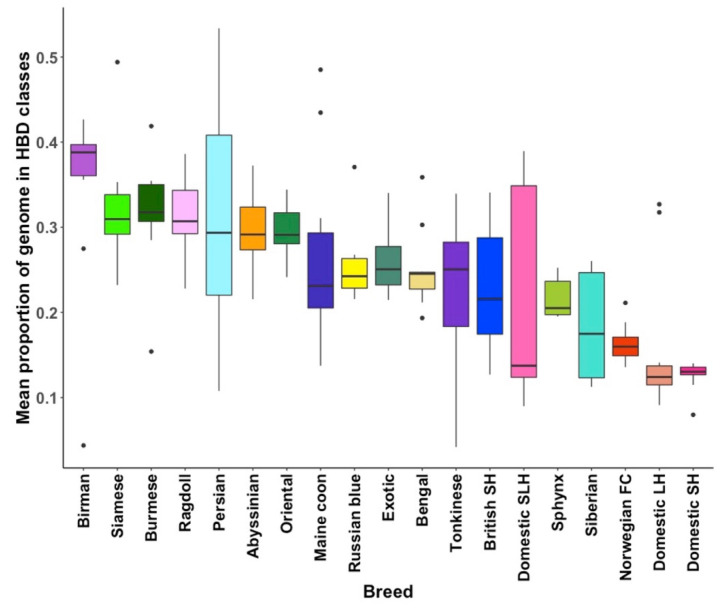
A boxplot diagram representing the mean proportion of each breed’s genome partitioned into HBD segments. On average, random-bred (i.e., DSH, DLH, DSLH) cats have the lowest proportion of their genome in HBD segments, whilst the Birmans had the highest proportion of their genome in HBD segments.

Homozygosity by descent (HBD) segment length is a function of generation time from initial inheritance, whereas the total genome content, classified by HBD, relates to levels of inbreeding [37]. Using the balanced dataset, the study observed that most pedigree cats have highly variable levels of both recent and older HBD segments (Figure 5). For pedigree cats, the mean genome content defined by HBD and hence inbreeding levels, were highest amongst Birman and Burmese cats (Figure 6). The Birman is the only population assessed where 9 of the 10 sampled cats had over 30% of their genome in HBD segments. In contrast, Norwegian forest cats had proportionally less of their genome defined by HBD segments compared to other pedigree cats. Those segments that were defined were mostly old (R_k_ = 512). Most pedigree cats have at least 10–20% of their genome in HBD segments that are rated 8–16 generations old.

**Figure A7:**
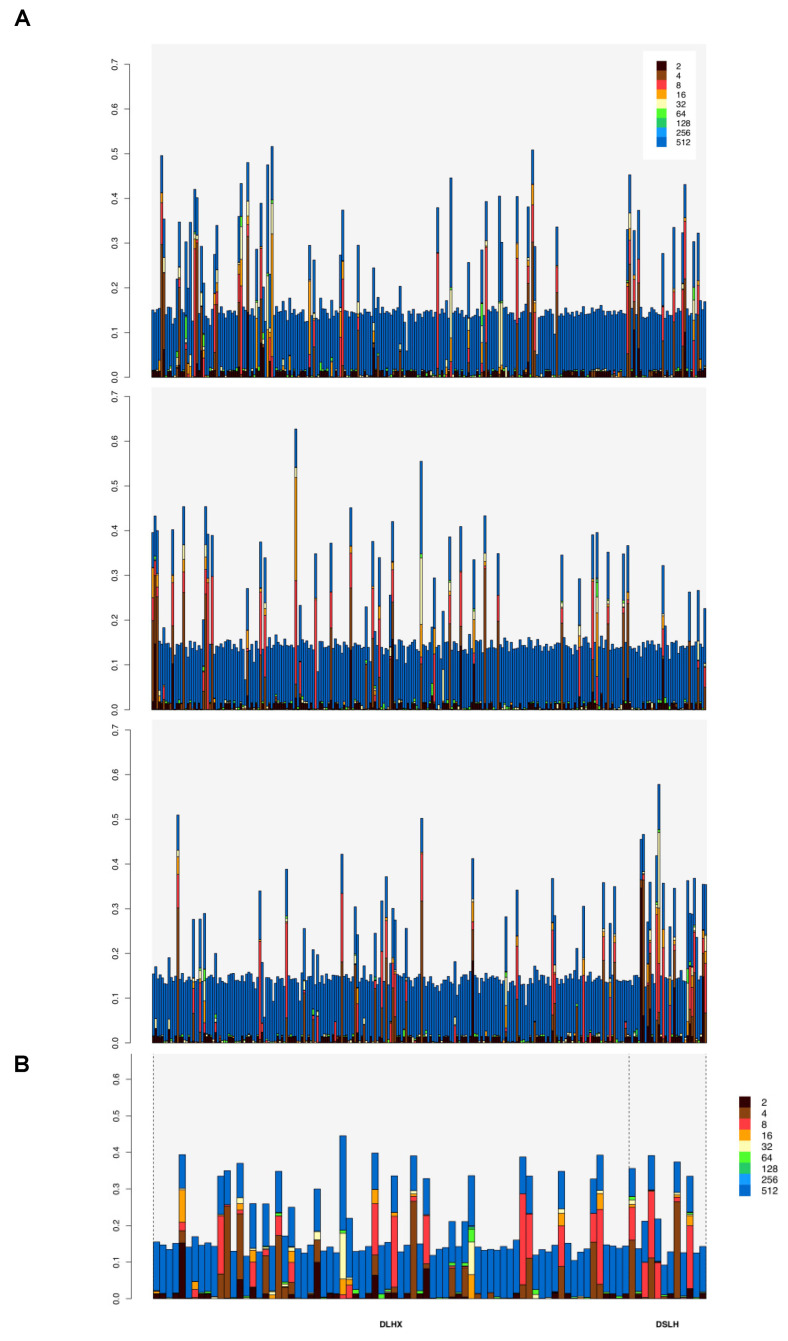
Genomic content of homozygosity by descent (HBD) segments domestic cats. HBD segments were classified by segments’ ages with respect to inferred generation time. The colour legend indicates the HBD class, defined by R_k_, to reflect the inheritance of HBD from common ancestors across many generations. (**A**) Genomic content of HBD segments for DSH cats (*n* = 754) and (**B**) of the DLH cats (*n* = 74) cats and DSLH cats (*n* = 12).

**Figure A8:**
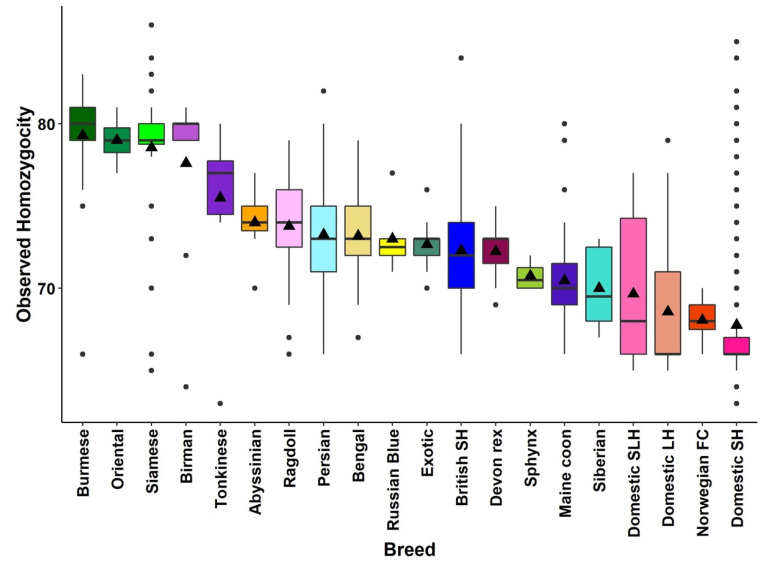
The observed homozygosity of all samples in the full dataset, only showing breeds included in the balanced dataset.

At roughly 12% of their genome, DSH cats have the least amount of HBD. Moreover, their HBD was old. Unexpectedly, 6 of the 30 random-bred cats showed evidence of recent inbreeding, as indicated by higher proportions of newer HBD segments. To investigate this further, all DSH, DLH, and DSLH cats were considered. In total, 19% (*n* = 840) had substantially newer HBD segments (Figure A6). To rule out a sampling artefact, we verified that the inbred cats originated from multiple sources located in both Scotland and England. The percent observed homozygosity was investigated for both the balanced dataset (Figure A7) and the full dataset (Figure A8). Results for the balanced dataset ranged from 63–84% observed homozygosity, with the highest mean observed homozygosity for the Siamese at 79% and Orientals at 78.5%, whilst the DSH and the DLH had the lowest mean observed homozygosity at 65% and 67%, respectively.

**Figure 7:**
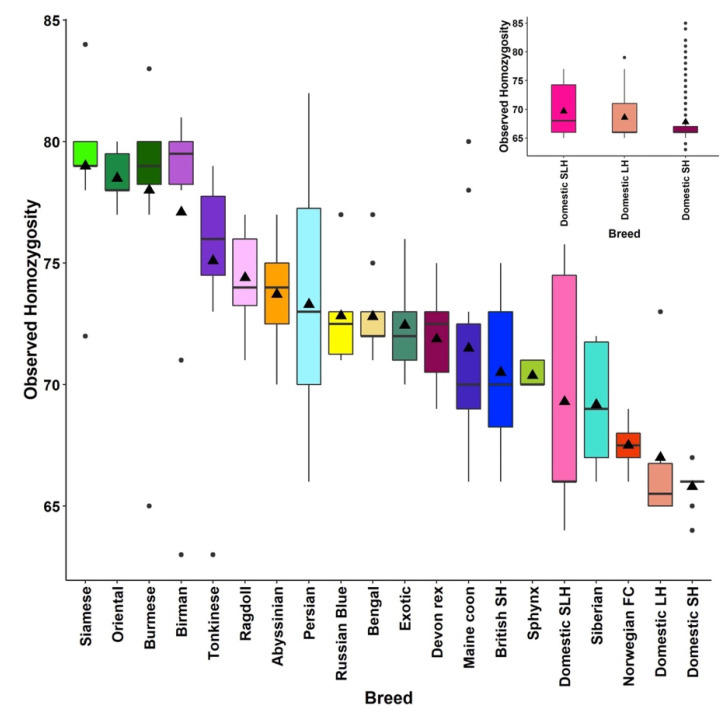
A boxplot diagram, representing the observed homozygosity for the balanced dataset, the range for the random-bred cats (i.e., DSH, DLH, and DSLH) is 64–76%. When all random-bred cats in the full dataset are examined, the range of observed homozygosity is 63–85% (inset). The black triangle symbol represents the mean observed homozygosity.

However, when the study looked at the individual ranges for observed homozygosity in the full dataset, the DSH had a range of 63–85%, indicating that some of the individual random-bred cats have observed homozygosity comparable with or even exceeding that of pedigree cats (Figure 7 inset).

## 4. Discussion

Despite rapid improvements in genome assemblies and genotyping technologies, their application to study non-pedigree cats remains historically limited. Here we used a custom SNV array composed of a dense probe set that was designed with random-bred cats in mind. We tested the array’s performance on 1344 UK-based cats whose composition roughly reflected the reality of cat ownership in the UK: over 75% of the cats in our study were non-pedigreed.

The current study is the first to look at the population genetics of this group of cats in the UK. This study confirms that these cats are a random-bred, rather than admixed population whose ancestry can be traced to pedigree animals. Moreover, although UK random-bred cats are in aggregate, genetically diverse, we observe striking examples of recent inbreeding despite sampling across the UK.

### 4.1. Breed Identification

Correct breed assignment is essential for the health and welfare of domestic cats for four main reasons. Firstly, various diseases are breed specific and knowing the correct breed may move a possible diagnosis further up or down the differential diagnosis list; this may ultimately affect the cat’s prognosis. Secondly, correct breed assignment is essential in genomics research to prevent false positives when mapping feline traits and diseases. Thirdly, breed is an integral part of legally identifying a cat, for example, on animal health certificates or pet passports. The fourth and final reason that the correct assignment of breed is important is for the owners’ interest; cat owners being one of the most passionate groups of animal owners, and knowing the breed and the breed history may help owners’ to pick the right breed for their lifestyle, it may even help them to bond with their cats.

There were rare cases in our PCA and fastSTRUCTURE analysis when cats with an assigned breed presented in clusters of another breed. For example, in our PCA, where two Birman cats present with the Ragdoll cluster, and a Maine coon cat presents with the Birman cluster. This can be observed in our fastSTRUCTURE analysis, where results support the PCA findings for these individual cats. Reasons for cats clustering with a different breed can include incorrect breed assignment or cryptic admixture. To investigate breed assignment, it is essential to know where the samples came from. The samples used in this study were obtained through veterinary practices and veterinary diagnostic laboratories. The breed recorded is usually based on owner reporting of the breed when registering at a veterinary practice. Unintentional breed misrepresentations may occur, where a particular breed is written down in error, or the owner thinks their rescued DLH cat is a Persian, for example. However, there are also cases of intentional breed misrepresentation. The breeding and sale of cats report [42] describes evidence of breed misrepresentation amongst cat sales.

Grouping non-pedigree cats by their hair length (DSH, DLH, and DSLH) is a common method of identification and is how they are registered in veterinary practices in the UK, as they do not have an official “breed”. These groupings were analysed to give complete clarity on the non-pedigree cats used. However, the DSH, DLH, and DSLH cats are effectively the same population, with autosomal recessive mutations in the fibroblast growth factor 5 gene *(FGF5*) being the primary cause of pelage length differences [10,11].

### 4.2. The UK Population Compared to Previous Studies

Despite different genotyping platforms and population sampling, the results from the current study recapitulate earlier breed relationships made by Lipinski et al. [5] and Gandolfi et al. [9]. The array resolved breeds with Southeast Asian, Western, and Persian origins. The current study also shows the distinctiveness of the Birman breed. Elsewhere, this uniqueness was attributed to genetic profile derived from a single extended Birman pedigree; however, the cats used in the current study had no discernible familial relationships (Figure A1). Moreover, the PCA appears to support its relationship with the Ragdoll, which is purportedly derived from crosses with the Birman [43].

Using fast STRUCTURE for the balanced dataset, at K = 11, this study was unable to distinguish the Maine Coon, Norwegian forest cat and the Siberian cat from each other, as well as from DSH, DLH, and DSLH. These cats are all Western origin cats. One anecdote of Maine Coon origins involves crosses between North American DSH and longhaired cats transported by Vikings [44]. Taylor and Negus [45] describe the Siberian as one of the possible precursors to the Norwegian forest cat breed. It is worth noting that both breeds are thought to be recently developed from Old World random-bred cats. Unexpectedly, the balanced dataset in the current study was unable to assign the British shorthair and Persian to individual groupings; rather, these breeds appeared admixed with the DSH, DLH, and DSLH. Perhaps this observation is indicative of the former’s UK origins and an underlying relationship with local random-bred cats. As noted elsewhere, we were unable to distinguish Oriental and Siamese cats due to their close ancestry [9].

Quantification of homozygosity offers insights into breed history and expansion. In general, all pedigree cats had elevated levels of HBD, as would be expected of breeding programmes involving small populations and the use of line breeding. The Ragdolls had one of the highest proportions and most uniform distributions of HBD segments. This could be interpreted as evidence of this relatively young breed’s small breeding population. The Persian’s mean HBD was similar, however individual HBD differed dramatically from sample to sample, a result that suggests some of the Persians sampled in this study were line bred. Norwegian forest cats, whose ancestry with random-bred cats is discussed above, displayed the smallest proportion of HBD segments, the majority of which were old. Although Southeast Asian and Eastern breeds such as the Birman and Siamese are among the oldest varieties of cats, these breeds are derived from small foundation stocks in the UK. The genomes of these breeds bore historic evidence a limited gene pool, as observed homozygosity was the highest among these breeds.

### 4.3. What Are the UK’s Non-Pedigree Cats?

In the strictest sense, any domestic animal that is not registered with a breed club is a ‘non-pedigree’ animal. Nonetheless, distinctions among ‘non-pedigree’ animals exist, as exemplified by so-called semi-feral village dogs: animals from the America’s are mostly breed admixtures, whereas village dogs from various regions within Africa are mostly local/indigenous admixtures [46]. The current study wished to investigate the genetic nature of the UK’s non-pedigree cats further. The fastSTRUCTURE results in this study showed that 91% of 840 non-pedigree cats formed a single cluster at K = 2; when analysed jointly with pedigree cats, the non-pedigree cats showed little evidence of pedigree cat admixture. From these results, we conclude that the UK’s non-pedigree cats are random-bred cats that populated the UK prior to breed formation. While the UK’s earliest evidence of domestic cats dates to the Iron Age, trade and conquest from Romans also brought domestic cats to the British Isles [47,48,49]. Curiously, our PCA results indicate that DSH, DLH, and DSLH are scattered across PC1 and PC2 axes, despite the absence of obvious introgression from Persian and Southeast Asian breeds. This paradox might be explained by an influx of random-bred cats from Persia and Southeast Asia to the UK prior to breed formation.

### 4.4. Health Implications for the UK’s Non-Pedigree Cats

The HBD analysis displays a wide variety of homozygosity within breeds. As a group, DSH, DLH, and DSLH cats are more genetically diverse than pedigree cats. However, when they are considered individually, the homozygosity of some of these random-breds is similar to or exceeds that of pedigree cats.

Unexpectedly, the current study observed a high incidence of long and young HBD segments among nearly 20% of the DSH, DLH, and DSLH cats that had been sampled. This observation is indicative of high levels of inbreeding and disputes previous work that suggests feral female cats avoid copulating with their close kin [50]. The reasons for this observation require further exploration; however, one possibility is that some UK DSH, DLH, and DSLH cats are produced in isolated settings, such as farms, or even cities where roads create boundaries preventing roaming. In these settings, copulation occurs between kin due to the isolation and lack of non-kin mating opportunities. Inbreeding depression can cause increased prevalence of genetic diseases as well as a reduction in fertility, longevity, and increased mortality [51].

### 4.5. SNV Array Performance

Population size and composition, heritability, and LD are critical factors that influence the success of genome-wide association studies (GWAS). Equally, ascertainment bias in SNV array design can distort population relationships, reduce the informativeness of markers, shift the frequency spectrum towards common alleles, and adversely influence population descriptors including variability, population structure, and recombination [52]. SNVs for the Hill’s Pet Nutrition SNV arrays were selected from whole genome sequencing of 6 genetically diverse female domestic shorthair cats. For random-bred cats whose LD decays rapidly, denser marker sets will be required to tag haploblocks [53]. The current commercially available 63 K feline SNV array has an average marker distance of 38 kb across the array and provided approximately >59 K informative, high-quality SNVs [9]. In contrast, the Hill’s Pet Nutrition SNV array provides approximately 143,000 SNVs (an average of 1 marker per 20 Kb). However, the informativeness of markers may differ from random-bred populations originating elsewhere. For these reasons, even denser genotyping arrays or genotype-by-sequencing may be preferable for conducting studies on random-bred cats, equally for pedigree varieties such as the Norwegian forest cat, whose LD decay was comparable to random-breds.

Overall, the UK’s DSH, DLH, and DSLH cats are a random-bred, genetically diverse cat population. However, as this study reveals, summary statistics can present an incomplete picture: unexpectedly, 19% of random-bred cat genomes displayed strikingly high proportions of HBD segments that is interpreted to result from recent and widespread inbreeding events. While DSH, DLH, and DSLH cats as a whole are genetically diverse, they are not impervious to inbreeding and, by extension, its health risks, including those caused by homozygous coupling of deleterious, recessive alleles.

## Figures and Tables

**Table 1:** Post-genotyping quality control. Six filtering steps were applied to remove duplicate samples as well as single nucleotide variants (SNVs) and samples with poor genotyping performance. MAF = minor allele frequency. PLINK v1.90b commands are in round brackets.

Quality Control Process	Genotyping Rate	Variants	Samples
Raw genotypes	0.95	263,482	1344
Remove duplicate samples: 34 cats	0.95	263,482	1310
1st pass remove variants (--geno 0.2): 14,257 SNVs	0.95	249,225	1310
1st pass remove samples (--mind 0.2): 9 cats	0.99	249,225	1301
2nd pass remove variants (--geno 0.02): 32,427 SNVs	0.99	216,798	1301
2nd pass remove samples (--mind 0.05): 11 cats	0.99	216,798	1290
MAF <= 0.05 (--maf 0.05): 38,292 SNVs removed	0.99	178,506	1290
**Analysis-ready genotype callset**	**0.99**	**178,506**	**1290**

**Table 2:** Cat breeds and their sample numbers in the cleaned dataset.

Breed	Number of Samples	Accepted Date of Origin	GCCF 2020	MAF	H_o_	F_IS_	Monomorphic SNVs	Informative SNzVs	Area of Origin
**Southeast Asia**									
Balinese	2	1940s	26	0.27	0.21	−0.216	61.71%	38.29%	USA
Burmese	27	1350–1767	1091	0.27	0.19	0.073	24.93%	75.07%	Thailand
Korat	1	1350–1767	42	0.25	0.18	N/A	82.05%	17.95%	Thailand
Oriental	6	1950s	619 ^1^	0.27	0.20	−0.035	45.08%	54.92%	UK
Siamese	36	1350–1767	1631	0.27	0.20	0.088	19.83%	80.17%	Thailand
Tonkinese	10	1950s	169	0.27	0.23	−0.042	32.28%	67.72%	USA
**Mediterranean**									
Abyssinian	7	1860s	143	0.25	0.24	−0.017	32.66%	67.34%	Ethiopia
Egyptian Mau	2	Early	58	0.25	0.25	−0.06	45.93%	54.07%	Egypt
Somali	2	1967	981	0.25	0.24	−0.211	56.54%	43.46%	USA/Canada
Turkish angora	1	Early	N/A	0.26	0.30	N/A	69.68%	30.32%	Turkey
Turkish van	2	Early	8	0.25	0.22	−0.009	48.06%	51.94%	Turkey
**Western**									
American shorthair	1	1900	N/A	0.25	0.32	N/A	67.94%	32.06%	USA
Cornish rex	2	1950s	71	0.26	0.27	−0.208	49.00%	51.00%	UK
Devon rex	8	1960s	321	0.25	0.26	−0.04	29.47%	70.53%	UK
Domestic longhair	74	N/A	N/A	0.25	0.29	0.077	10.05%	89.95%	UK
Domestic semi-longhair	12	N/A	N/A	0.25	0.28	0.066	16.34%	83.66%	UK
Domestic shorthair	754	N/A	N/A	0.24	0.30	0.058	8.17%	91.83%	UK
Maine coon	47	1860s	2566	0.25	0.27	0.061	13.66%	86.34%	USA
Manx	2	Early	21	0.25	0.28	N/A	44.88%	55.12%	UK
Norwegian forest cat	15	Early	301	0.25	0.30	−0.001	17.24%	82.76%	Norway
Siberian	6	Early	358	0.25	0.28	−0.001	24.53%	75.47%	Russia
Sphynx	8	1966	196	0.25	0.27	−0.04	27.23%	72.77%	Canada
Tiffany	1	1967	N/A	0.26	0.24	N/A	75.58%	24.42%	USA
**Persian**									
Persian ^2^	51	Early	908	0.24	0.25	0.113	14.77%	85.23%	Iran
Persian/Western									
British shorthair	95	1870’s	9111	0.24	0.26	0.096	11.70%	88.30%	UK
Exotic shorthair	15	1966	350	0.25	0.25	−0.017	29.09%	70.91%	USA
**Southeast Asia/Persian**									
Asian	2	1981	187 ^3^	0.26	0.20	−0.163	61.35%	38.65%	UK
Birman	10	1930s	472	0.26	0.21	0.024	30.24%	69.76%	Burma
**Hybrid**									
Bengal	27	1963	263	0.26	0.25	0.082	16.96%	83.04%	USA
Savannah cat	1	1997	N/A	0.26	0.28	N/A	71.83%	28.17%	UK
**Eastern/Western**									
American bobtail	1	1960s	N/A	0.25	0.32	N/A	68.29%	31.71%	USA
Bombay	1	1958	N/A ^4^	N/A	0.20	−0.5	79.73%	20.27%	USA
Ragdoll	27	1960s	4387	0.25	0.24	0.056	19.26%	80.74%	USA
Russian blue	6	Late 1800s	443	0.25	0.25	−0.071	35.77%	64.23%	Russia
Selkirk rex	1	1987	89	0.24	0.26	N/A	73.55%	26.45%	USA
Snowshoe	2	1960s	73	0.26	0.29	−0.16	44.85%	55.15%	USA
**Unknown**									
Cross	17	N/A	N/A	N/A	N/A	N/A	N/A	N/A	N/A
Unknown	8	N/A	N/A	N/A	N/A	N/A	N/A	N/A	N/A
Total	1290								

Accepted date of breed origin and area of origin, sourced by Shartwell, J, Moarace, T and Shelton, L as cited in Vella et al. [7] except in the case of the Asian, sourced from the General Council for the Cat Fancy (GCCF) [38]. N/A; not applicable. To give an estimate of breed population size in the UK, numbers of cats registered with the GCCF in 2020 are provided in the column: GCCF 2020. Breed average minor allele frequency (MAF), breed average observed heterozygosity (H_o_) and breed average inbreeding coefficient (F_is_) prior to MAF filtering are also included. Breeds are mostly assigned to one of four main groups for ease of discussion. “Southeast Asia” to classify Asian origin breeds, “Mediterranean” to classify Mediterranean basin origin breeds and “Western” to classify Western European and USA origin breeds. “Persian” reflects this breed and its derivatives. Some breeds are derived from a combination of the aforementioned categories or from a transitional zone. Owing to their recent ancestry with wild felids, Bengal, and Savannah cat are classified as “Hybrids”. ^1^ Shorthair. ^2^ Including Chinchilla Persians. ^3^ Shorthair and longhair. ^4^ Included in Asian registrations.

**Table 3:** A second dataset was extracted from the original clean dataset, containing only breeds with more than 5 samples, and for those breeds with more than 10 samples, the R function “sample_n” was used to select 10 samples at random from the population.

Breed	Number	MAF	H_o_	F_IS_	Monomorphic SNVs	Informative SNVs
Abyssinian	7	0.25	0.24	−0.017	32.66%	67.34%
Bengal	10	0.25	0.25	−0.020	29.17%	70.83%
Birman	10	0.23	0.21	0.024	30.24%	69.76%
British shorthair	10	0.25	0.27	0.030	21.07%	78.93%
Burmese	10	0.23	0.20	0.031	32.22%	67.78%
Devon rex	8	0.24	0.26	−0.040	29.47%	70.53%
Domestic longhair	10	0.26	0.30	0.006	17.62%	82.38%
Domestic shorthair	10	0.26	0.31	−0.036	17.85%	82.15%
Domestic semi-longhair	10	0.26	0.28	0.059	17.55%	82.45%
Exotic	9	0.25	0.25	0.017	29.09%	70.91%
Maine coon	10	0.25	0.26	0.082	20.54%	79.46%
Norwegian forest cat	10	0.26	0.30	−0.009	19.41%	80.59%
Oriental	6	0.23	0.20	−0.035	45.08%	54.92%
Persian	10	0.25	0.25	0.108	22.28%	77.72%
Ragdoll	10	0.24	0.23	0.005	31.63%	68.37%
Russian blue	6	0.25	0.25	−0.071	35.77%	64.23%
Siamese	10	0.22	0.19	0.026	38.07%	61.93%
Siberian	6	0.25	0.28	−0.001	24.53%	75.47%
Sphynx	8	0.25	0.27	−0.040	27.23%	72.77%
Tonkinese	10	0.23	0.23	−0.042	32.28%	67.72%
**Total**	**180**					

Breed average minor allele frequency (MAF), breed average observed heterozygosity (H_o_), and breed average inbreeding coefficient (F_is_) prior to MAF filtering are also included.

## Data Availability

The data presented in this study are openly available from Edinburgh DataShare at https://doi.org/10.7488/ds/3072 (accessed on 12th October 2021).
